# Determinants of Equitable Data Governance for African, Caribbean, and Black Communities in Health Research in High-Income Countries: Protocol for a Scoping Review

**DOI:** 10.2196/82403

**Published:** 2026-02-13

**Authors:** Josephine Etowa, Shamara Baidoobonso, Doris Kakuru, OmiSoore Dryden, Chinedu Oraka, Egbe Etowa, Peter Farrell, Sandra Mba, Akalewold Gebremeskel, Arone Wondwossen Fantaye

**Affiliations:** 1 School of Nursing Faculty of Health Sciences University of Ottawa Ottawa, ON Canada; 2 Chief Public Health Office Government of Prince Edward Island Charlottetown, PE Canada; 3 School of Child and Youth Care Faculty of Education University of Victoria Victoria, BC Canada; 4 Faculty of Medicine Dalhousie University Halifax, NS Canada; 5 Canadians of African Descent Health Organization (CADHO) Ottawa, ON Canada; 6 School of Nursing Toronto Metropolitan University Toronto, ON Canada

**Keywords:** African, Caribbean, Black community, data governance, data ownership, scoping review protocol

## Abstract

**Background:**

African, Caribbean, and Black (ACB) communities in high-income countries continue to experience persistent health inequities, driven by systemic anti-Black racism, socioeconomic disadvantage, and exclusion from health decision-making. Historically, data have been extracted from ACB communities without transparency, accountability, or community ownership. These inequitable practices have produced data systems that reinforce harm rather than promote equity. Equitable data governance, which promotes community ownership over data collection, access, and use, is increasingly recognized as a critical but underresearched determinant of health equity.

**Objective:**

This protocol outlines the methodology of a scoping review to identify and synthesize evidence on the determinants of equitable data governance in health research involving ACB communities in high-income countries.

**Methods:**

The review follows the 6-stage Arksey and O’Malley methodological framework, supplemented with updated guidance from the Joanna Briggs Institute. The searches were conducted in the Ovid MEDLINE, Ovid Embase, EBSCO CINAHL, APA PsycInfo, and Scopus databases. Peer-reviewed articles are considered, with no limits placed on study design, publication type, or date. Multiple reviewers will independently extract data by using a standardized form. A 3-phase thematic mapping process, conceptually informed by critical race theory, intersectionality, and community-based participatory research principles, will be conducted to analyze the data, generate themes, and interpret findings.

**Results:**

The final comprehensive database searches were completed on December 17, 2024. The search strategy targeted literature on data management, governance, sharing, security, and ethical principles in relation to ACB populations in high-income countries. A total of 4365 records were screened at the title and abstract level, after deduplication, of which 247 studies were deemed potentially relevant and advanced to full-text screening. Following full-text screening and reference list searching a total of 15 articles were deemed eligible for analysis. The data extraction stage is scheduled to overlap and occur between November 2025 and February 2026. The thematic mapping and stakeholder consultations processes are scheduled between December 2025 and February 2026. The final review and manuscript submission are expected by March 2026, with dissemination activities planned for mid-2026.

**Conclusions:**

This review will synthesize existing information on key pillars, barriers, facilitators, promising data governance policies and practices, and recommendations relevant to ACB communities. The findings may inform the expansion of Ontario’s Engagement, Governance, Access, and Protection guidelines and support tailored research and national data governance frameworks. The review is expected to contribute to policy, research, and community-led data initiatives. Dissemination will occur through academic publications, conferences, and community-based knowledge-sharing events. As the review relies solely on publicly available data, ethics approval is not required.

**Trial Registration:**

OSF Registries 10.17605/OSF.IO/Z82AY; https://osf.io/z82ay

**International Registered Report Identifier (IRRID):**

PRR1-10.2196/82403

## Introduction

Persistent health inequities in high-income countries (HICs) disproportionately affect African, Caribbean, and Black (ACB) communities, contributing to poorer health outcomes among the population despite positive health behaviors [1,2]. These inequities are notable in the prevalence of chronic conditions such as diabetes, HIV, and most recently COVID-19 [3-6]. The literature indicates that health outcomes are driven by a range of upstream factors, such as historical injustices, socioeconomic disadvantage, and systemic anti-Black racism [7,8]. These inequities stem from historical and structural injustices, including slavery, colonization, and intersecting oppressions, such as sexism, classism, and xenophobia, that mold the lived experiences of ACB communities and perpetuate racial health disparities. Anti-Black racism is rooted in societal structures and contributes to systemic inequalities across sectors such as education, employment, housing, health care, and criminal justice [9]. Its cumulative effects, which include microaggressions and institutional discrimination, have a significant impact on health access, uptake, and outcomes.

Awareness of racial inequities in health has grown, but efforts to address them are often impeded by inequitable data systems, policies, and practices [10,11]. Data shape all aspects of contemporary existence, informing policy development and determining what the world around us looks like [12-14]. The availability, collection, access, and use of health data can either remove or reinforce structural barriers and health inequities in communities. Historically, data have been extracted, manipulated, and deployed to justify and bolster slavery, violence, and anti-Black racism [15-17]. ACB communities have also historically been excluded from decisions about how their health data are collected, protected, interpreted, and used [16,17]. Such inequitable data governance practices persist today, as data continue to be extracted from ACB communities without transparency, accountability, or adequate community engagement [18,19]. In addition, ACB research leaders and ACB-led organizations remain underrepresented in governance structures and decision-making processes that address ACB community concerns [1,20]. It is more common for ACB communities to be engaged primarily as participants or community partners, rather than being positioned as leaders of research within their own communities.

There is growing acknowledgment that ACB communities, ACB-led organizations, and scholars need to be involved in efforts to address health issues that directly affect them [21]. Empowering and involving ACB communities and ACB-led organizations in data systems can nurture trust, strengthen community data ownership, and enhance access to community cultural knowledge and insights that can inform and enrich research [22]. In addition, policies and programs intended to address health issues will be better informed and aligned with the needs and priorities of the communities themselves. The cultivation of community-oriented and equitable data governance is thereby imperative for facilitating equitable data practices that protect against misuse and support self-determined health solutions.

The COVID-19 pandemic and the rise of digital health technologies have intensified attention to the topic of data governance. Increasingly, data governance approaches centering on marginalized populations in health research are recognized as mechanisms for the translation of data into targeted interventions and medical technologies that can be used by both community members and researchers to drive positive health outcomes. The process can bring about transformation, equity, and social justice within health care systems [23]. HICs have demonstrated this growing momentum through the development of inclusive, transparent, and community-led data models and frameworks. For instance, in Canada, the First Nations Information Governance Centre (FNIGC) developed the Ownership, Control, Access, and Possession (OCAP) principles as an expression of self-determination in research [24]. The FNIGC developed strategic partnerships for unique data-gathering initiatives and built data and statistical capacities at national, regional, and community levels for the provision of credible and relevant information on First Nations.

The development of OCAP inspired the creation of the Engagement, Governance, Access, and Protection (EGAP) framework, focused on data management and community governance to address systemic racism and advance health equity in ACB communities [16]. On the basis of the learnings from OCAP, EGAP’s guidelines envision ACB communities gaining control over their collective data through community governance tables, which are decision-making bodies that approve or reject plans for community engagement, data collection, and analysis [25]. Similar calls for community-centered governance have emerged internationally. A recent US study advocating for data governance among Black patients with breast cancer pointed to the urgent need for them to have better access to health data, genetic counseling, education, and inclusion in research [23]. In the United Kingdom, key health care leaders involved in the UK-REACH study acknowledged the importance of minority groups having access to health research data to drive better outcomes [14]. Specifically, they identified a need for “Big Data Ethics by Design” to promote community engagement within health research and in turn drive scientific breakthroughs that can be translated into medical innovation and effective public health intervention [14]. Collectively, these examples illustrate how community-led governance can transform data from a tool of harm into a mechanism for equity and justice.

Despite conceptual arguments and promising recent developments in data governance, there remains a lack of synthesized knowledge on what constitutes equitable data governance for ACB communities in health contexts in HICs. This protocol outlines the methodology for a scoping review that addresses this gap by exploring and synthesizing the evidence on the determinants, components, and promising policies and practices that support equity in data governance for ACB communities in HICs. The review is timely and necessary and will inform future research, policy, and practice to support racial health equity through inclusive, ethical, and community-centered data governance. The evidence from this review can help lay the groundwork for improved health research outcomes and promote health equity in ACB communities.

## Methods

### Approach

This protocol was registered to the Open Science Framework registry. The proposed scoping review is being conducted according to the 6-stage scoping review methodological framework by Arksey and O’Malley [26], which includes (1) identifying the research questions; (2) identifying relevant studies; (3) selecting the studies; (4) charting the data; (5) collating, summarizing, and reporting the results; and (6) consulting experts and stakeholders. Guidance from the Joanna Briggs Institute (JBI) *Manual for Evidence Synthesis* is being integrated to enhance methodological rigor and address known limitations of the earlier scoping review practices [27-29]. Specifically, the reviewers draw on JBI’s guidance for the development and piloting of the data extraction tool (stage 4), the approach to the synthesis and presentation of the results (stage 5), and incorporation of stakeholder consultation as an iterative and reflexive process (stage 6). This combined approach enables us to adhere to foundational scoping review methodology and align with current best practices in evidence synthesis. This protocol is reported in accordance with the PRISMA-P (Preferred Reporting Items for Systematic Reviews and Meta-Analysis Protocols) guidelines.

### Step 1: Identifying the Research Question

In determining the research questions, this scoping review used the population, concept, and context framework to fully examine the scope and gaps within the current literature. Textbox 1 provides key definitions of the population (ACB), concept (data governance), and context (HIC) selections.

**Definitions of key concepts used in this scoping review.**

- African, Caribbean, and Black: refers to individuals and communities who identify as Black and trace their heritage to sub-Saharan Africa, the Caribbean, or the African diaspora globally. We use this term inclusively of Black people from diverse cultural, ethnic, and national backgrounds.
- Data governance: refers to data systems, structures, policies, and practices that determine who has influence and control over the agenda, data collection, ownership, interpretation, and application of health data [16,17]
- High-income countries: listed using the 2023 World Bank classification and Organization for Economic Co-operation and Development country membership [30]

Textbox 2 details the primary research question and secondary research questions. For secondary research question 1, *promising* policy and practices are conceptualized as innovative policies and practices that show potential to improve equity in data governance, even if not yet widely implemented, adopted, or evaluated [31,32]. For secondary research question 2, “effective and comprehensive” are being conceptualized as tools, frameworks, or practices that demonstrate evidence of improvement in health equity outcomes, legal and ethical safeguards, community trust and uptake, mechanisms for community engagement, and increased ACB community power over data structures and processes [33]. These components are considered comprehensive when they address multiple levels (eg, policy, organizational, and community) or dimensions of governance, such as ownership, access, protection, use, and accountability.

**Research questions.**

**Primary research question**

- What are the determinants of equitable data governance (concept) for African, Caribbean, and Black (ACB) communities (population) in health contexts in high-income countries (context)?

**Secondary research questions**

- What are promising policies and practices to address equity in data governance for ACB communities and ACB-led organizations in high-income countries? (secondary research question 1)
- What are the components of effective and comprehensive data governance frameworks to strengthen ACB communities’ health in high-income countries? (secondary research question 2)

### Step 2: Identifying Relevant Studies

A highly sensitive multidatabase search strategy was developed, using controlled vocabulary and text words to identify peer-reviewed literature that touched on data governance in relation to research on or with diasporic Black populations with African or Caribbean roots in HICs. The following databases were searched by a health sciences librarian (PF): MEDLINE All via Ovid, Embase + Embase Classic via Ovid, APA PsycInfo via Ovid, CINAHL via EBSCOhost, and Scopus. A search strategy was developed in the MEDLINE database and then translated into the other databases, as appropriate (Multimedia Appendix 1). The MEDLINE database search strategy was peer-reviewed using the Peer Review of Electronic Search Strategies tool by another librarian (Marie-Cécile Domecq). All databases were searched from inception to December 17, 2024. The search will be updated before reporting the results. Following the screening of database results, the researchers will conduct forward and backward citation searching on the included articles.

To increase the breadth of the scoping review, source evidence was open to all peer-reviewed publication types, including primary studies, conceptual and opinion papers, secondary studies, reports, etc. Only English and French evidence sources were considered due to feasibility reasons, as they are the primary languages of the researchers. In addition, only evidence sources with a primary focus on ACB populations were considered so that issues specific to this target population are addressed.

### Step 3: Study Selection

The research team seeks to identify information sources that examine the factors that contribute to equitable data governance for ACB communities. Sources reporting on effective, comprehensive, or promising practices, policies, tools, and frameworks will also be considered. A summary of the inclusion and exclusion criteria for the primary and secondary research questions is detailed in Tables 1 and 2, respectively. In cases where articles focus on broader racialized or visible minority populations, inclusion will be contingent on the clear identification and description of ACB representation and experiences.

Source selection includes two stages: (1) title and abstract screening and (2) full-text screening. Both stages will use the software program Covidence, which will automatically remove duplicate articles. Four reviewers (AWF and volunteer students) are independently screening titles and abstracts, with disagreements being resolved through discussion with the senior author (JE). Full texts of potentially relevant studies will be independently screened by the same four reviewers. Disagreements between 2 full-text screeners will be resolved by a third screener in Covidence or by discussion with the senior author (JE) if needed by the third screener. The number of excluded articles and the rationale for exclusion will be recorded in the PRISMA-P scoping review flowchart.

**Table 1:** Eligibility criteria for the primary research question.

	Inclusion criteria	Exclusion criteria
Population	Includes any person or an individual, organizations, community, or stakeholders that are identified as ACB^a^ or related terms, and conduct research among the ACB population. Articles will be considered if they report on determinants of equitable data governance in racialized communities, if the ACB community is clearly mentioned and described	Articles that do not include ACB populations, clients, or patients
Concept	The concept of data governance between researchers, agencies, and other data governing bodies with ACB communities or ACB-led organizations. This can encompass data ownership, control, and access to improve health outcomes, as well as research partnerships, collaborations, and knowledge mobilization practices	Articles that are not relevant to data governance
Context	High-income countries listed in the 2023 World Bank classification and Organization for Economic Co-operation and Development country membership [30]	Articles that are not from a high-income country context (eg, articles from a middle- or low-income country)
Source type	Open	Full-text unavailable
Language	English and French	Full-text is only available in languages other than English and French.
Publication date	No date restrictions	—^b^

^a^ACB: African, Caribbean, and Black.

^b^Not applicable.

**Table 2:** Eligibility criteria for secondary research questions.

	Inclusion criteria	Exclusion criteria
Population	ACB^a^ communities and ACB-led organizations	Articles that do not include ACB populations, clients, or patients
Concept	Data governance policies and practices. These may include promising policy and practices that show potential to improve equity in data governance, even if not yet widely implemented, adopted, or evaluated. Effective and comprehensive data governance frameworks between researchers and ACB communities. May include tools, frameworks, or practices that demonstrate evidence of improvement in health equity outcomes, legal and ethical safeguards, community trust and uptake, mechanisms for community engagement, and increased ACB community power over data structures and processes	Articles that are not related to data governance policies and practices
Context	High-income countries listed in the 2023 World Bank classification and Organization for Economic Co-operation and Development country membership [30]	Articles that are not from a high-income country context (eg, articles from a middle- or low-income country)
Source type	Open	Abstracts, full-text unavailable
Language	English and French	Full-text is only available in languages other than English and French
Publication date	No date restrictions	—^b^

^a^ACB: African, Caribbean, and Black.

^b^Not applicable.

### Step 4: Charting Data

Data will be extracted from papers included in the scoping review independently by 4 coders (AWF, a senior reviewer, and 2 volunteer students) using a data extraction tool developed by the reviewers. The data will include specific details about the population, concept, context, study methods, and key findings relevant to the scoping review objectives. A draft of the extraction table is provided (Multimedia Appendix 2). This will be modified and revised as necessary during the extraction of the included studies. Modifications will be detailed in the full scoping review report.

To calibrate the table, all 4 coders will independently extract data from 3 evidence sources, which will subsequently be compared. Creating the table is an iterative process, and modifications may be made through the same process as more evidence emerges [34]. Any discrepancies between the coders will be discussed until at least a 75% agreement is achieved. To ensure consistency and catch discrepancies early on, the 4 coders will then independently extract from a subset of the remaining included articles (at least 25%). Two recalibration meetings will be held with the senior author (JE) after extracting approximately a quarter and then half of the articles to review and discuss extractions. Disagreements during subset extraction or recalibration meetings will be resolved through discussion during these meetings, with oversight by the senior author (JE).

The current template for this table contains (1) first author; (2) date; (3) country of origin; (4) population; (5) organizational resources, infrastructure, knowledge, and skills; (6) culture and climate; and (7) engagement and partnership, study design, sample size, findings, and conclusion. Given that several different types of evidence sources are anticipated, it is not expected that all categories in the general extraction table will be completed for each evidence source; not applicable will be written, where appropriate. However, the extracted data from each evidence source will align with the research question and objectives based on reviewing its entire text. Microsoft Word will be used during the extraction process to record and organize the data. The extracted information will be used to inform the collation and summarization of the findings from the evidence sources.

### Step 5: Collating, Summarizing, and Reporting Results

#### Overview

The data extracted from relevant published literature will be presented in a tabular form that is aligned with the objectives of this scoping review. Data that are presented in tables will reflect the information collected using the data extraction tool. A narrative summary will accompany the tabulated and charted results and will describe how the results relate to the review objectives.

**Figure 1:**
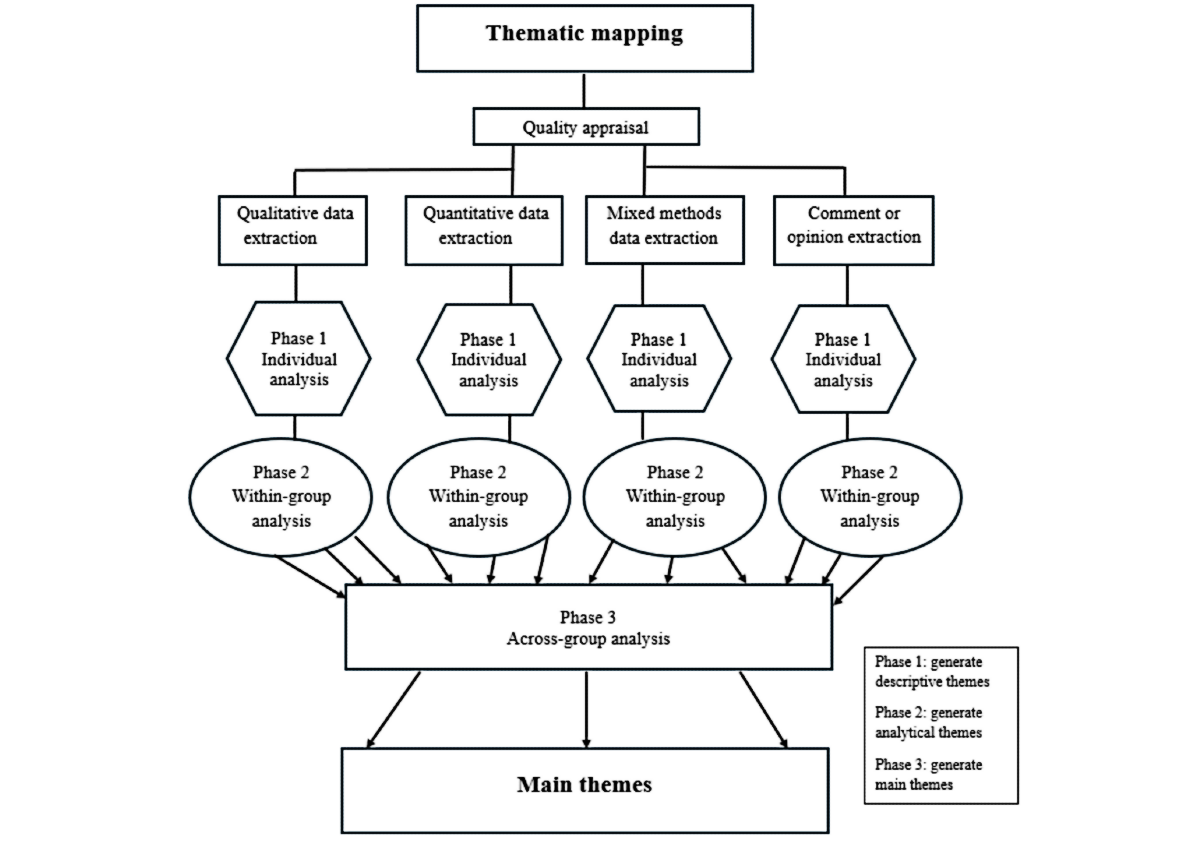
Thematic mapping process.

Thematic mapping (TM) [35] will be used to collate and summarize the extracted data, as it is ideal for accommodating the multiple types of evidence sources in this study (Figure 1) [21]. In addition, TM allows for the production of themes while maintaining the breadth of information within each theme through the creation of subthemes (descriptive and analytic themes). This approach is consistent with other methodological approaches from Arksey and O’Malley [26] and Peters et al [27,28] who acknowledge that results should be descriptively mapped rather than synthesized.

#### TM Phase 1: Individual Study Analysis

The aim of phase 1 will be to create initial codes and descriptive themes, informed by 3 conceptual underpinnings: critical race theory, intersectionality, and community-based participatory research. The critical race theory and intersectionality lenses will specifically be used as sensitizing concepts to sensitize the coders to the power dynamics, systemic inequities, and overlapping marginalization in data governance structures and processes. Community-based participatory research will also be used as a sensitizing concept to pull out the presence (or lack thereof) and consideration of community voice and priorities in the data. Together, they facilitate consideration of the cause and persistence of systemic racism and discrimination in the structure and processes through which data are collected, managed, analyzed, and used.

#### TM Phase 2: Within-Group Analysis

In phase 2, analytic themes will be created to gain further insight into the characteristics of the descriptive themes that reflected the content within each type of article grouping (qualitative, quantitative, mixed method, and opinion or conceptual) separately. The descriptive themes from each article will be grouped based on similarities and differences into analytic themes through induction and consistent interpretation with the population, concept, and context research questions, and the theoretical underpinnings, as in phase 1. The iterative process of creating the final analytic themes will be concluded when a consensus is reached between the research team members.

#### TM Phase 3: Across-Group Analysis

Main themes will be created to give a broad overview of findings based on the similarities and differences across all groups. These main themes will be used to map the evidence on concepts and boundaries, help identify research gaps, and determine existing strategies to dismantle the barriers causing ACB-led organizations to genuinely conduct and lead research. The themes that will be developed will be recorded in the thematic map.

### Step 6: Consultation

Scoping review results will be brought to the African, Caribbean, and Black National Expert Working Group (ACB-NEWG) in Canada for collaboration and input. ACB-NEWG is comprised of approximately 20 diverse stakeholder groups, including Black representatives from community-based organizations, Black community–engaged researchers, and Black representatives from mainstream organizations (eg, Public Health Agency of Canada), as well as coapplicants and collaborators. The ACB-NEWG will serve as the Project Advisory Committee for this project. Therefore, the executive group will review the findings and preliminary frameworks and provide feedback on evaluation items that may need to be added, subtracted, or altered. Having a broad Steering Committee, project advisory committee, and a network of potential partners, an effective communication strategy, and clear lines of responsibility will help to assure that the governance structure is sustained over the course of the project.

### Ethical Considerations

This manuscript does not report on or involve the use of any animal or human data or tissue.

## Results

**Figure 2:**
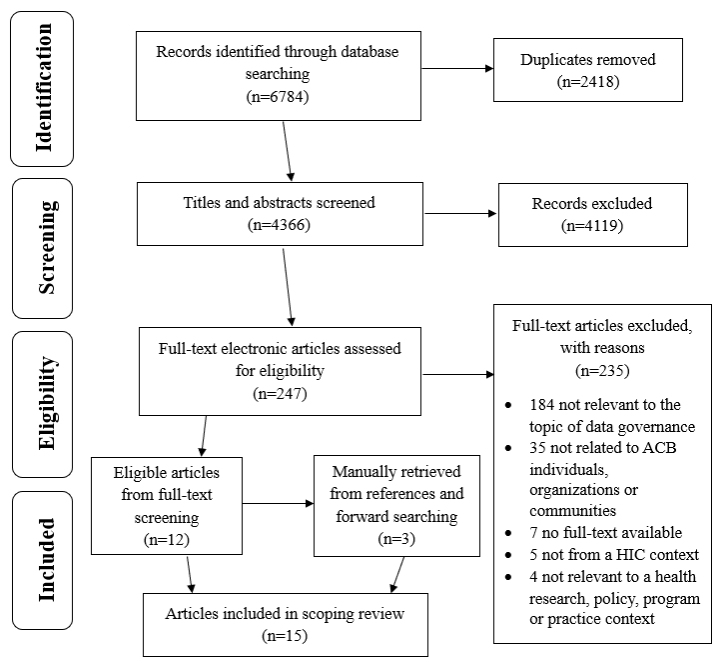
PRISMA (Preferred Reporting Items for Systematic Reviews and Meta-Analyses) flowchart of the identification of articles via databases and reference lists. ACB: African, Caribbean, and Black; HIC: High income country.

The comprehensive database searches have been completed across MEDLINE, Embase, CINAHL, APA PsycInfo, and Scopus, with the final searches run on December 17, 2024. A total of 6784 records were imported for screening. After removing 2418 duplicates (2414 via Covidence and 4 manually), 4365 records were screened at the title and abstract level. Preliminary observations from the abstract screening suggest that a diverse range of literature reports on determinants, frameworks, and promising policies and practices to empower and facilitate equitable and community-led data governance in racialized contexts, though the relevance and representation to ACB-led governance appear limited (Figure 2).

Only 247 records were identified as potentially eligible and have progressed to full-text screening, which assessed each article against the inclusion criteria outlined in Tables 1 and 2. This phase was completed in December 2025, along with manual reference list searching and screening. A total of 12 relevant articles have been retrieved from full-text screening, and 3 additional relevant articles from reference list searching. The data extraction stage has commenced and is scheduled to be completed by mid-February 2026. The TM process and final stakeholder consultations are scheduled to overlap and occur between December 2025 and late-February 2026. The final review and manuscript submission are expected by early March 2026, with dissemination activities planned for mid-2026.

## Discussion

### Anticipated Findings

This scoping review protocol outlines a rigorous and inclusive methodology for the synthesis of the existing information on equitable data governance in health research involving ACB communities in HICs. The findings will provide a broad perspective from a variety of evidence sources, with a focus on understanding the key factors that contribute to the inclusive, ethical, and participatory approach of data governance for ACB communities and how these contribute to improved outcomes. Moreover, it will also help identify promising practices, policies, tools, and frameworks that contribute to improved health outcomes for ACB communities. Full-text screening has revealed a diverse body of literature, although the representation of ACB-led initiatives remains limited. In multiple articles, ACB communities are represented within broader racialized or visible minority populations. For the purposes of comprehensiveness and relevance, such articles are being included in the review if they clearly identify and describe ACB representation and experiences. This approach allows us to capture insights from multiethnic contexts while maintaining a focused lens on ACB-specific data governance issues.

Frameworks such as OCAP and EGAP have laid the foundational principles for Indigenous and Black data governance in certain pockets of Canada. However, there is limited synthesis of how these principles are operationalized across health research involving ACB communities in HICs. This review will build on existing conceptual work by mapping existing information on determinants, policies, and practices, and identifying opportunities for enhancing data equity and community control over ACB health data. The findings may inform the refinement of EGAP and support the development of a broader and more equity-focused national data governance framework that can be adapted to the Canadian context and beyond, including other HICs where ACB communities face similar data governance, policy, and practice barriers. The review will also offer more practical insights into the policies, practices, and research that are needed to meet the needs of ACB populations, including enhancing their data governance capabilities.

### Strengths and Limitations

To limit bias, this review predefines the search question, objectives, methodology, eligibility criteria, search methods, data extraction techniques, summary, and the presentation of anticipated findings. This structured approach enhances transparency and reproducibility. The use of a peer-reviewed, multidatabase search strategy and a structured TM process further enhances methodological rigor and transparency. Another key strength of this review lies in its integration of critical race theory, intersectionality, and community-based participatory research, which provides a theoretically grounded and equity-focused review approach. Finally, stakeholders who are engaged throughout the review will be invited to review preliminary findings and thematic maps to ensure that the interpretations reflect community priorities and lived experiences.

However, multiple practical and operational limitations must be acknowledged. First, the concept of “equitable data governance” is still evolving and may be interpreted differently across studies and contexts. This could lead to difficulties in identifying and categorizing relevant determinants, practices, policies, or recommendations. Second, there may be limited representation of ACB-led data governance initiatives in the peer-reviewed literature, which could restrict the review’s ability to capture community-driven models, and existing promising policies and practices. Third, the language restriction to English and French may result in the exclusion of relevant studies published in other languages. Fourth, the inclusion of diverse evidence sources (primary research, reports, opinion, and conceptual papers) that are different in quality, depth, and format may make it difficult to extract consistent data. Despite the limitations, this scoping review will help to examine the evidence available and gauge the current state of the literature on factors influencing data governance for ACB communities and ACB-led organizations.

### Future Directions

The findings of this review may inform future empirical research on community-led data governance models, including longitudinal evaluations of their impact on health equity. Additionally, the review may uncover conceptual and methodological gaps, such as the need for standardized definitions, metrics for equity, and frameworks for the evaluation of governance effectiveness across diverse ACB contexts.

### Dissemination

The results from the proposed scoping review will be disseminated through peer-reviewed open-access journals and conferences targeting public health stakeholders, vaccination campaigns, and overcoming inequities in health care. In addition to academic dissemination, the reviewers will codevelop and share plain-language summaries of key findings with community partners and stakeholders, including members of the ACB-NEWG. These summaries will be tailored for nonacademic audiences and shared through partner networks, community knowledge-sharing events or workshops (eg, World Café events), and public health forums. Stakeholders engaged during the consultation phase will also advise on other culturally responsive and context-relevant formats for disseminating findings, with the aim of maximizing accessibility, relevance, and potential for policy or practice use within ACB communities.

## Data Availability

This protocol does not report on any datasets. Data generated during the review will be made available upon publication of the final review.

## Appendix

Multimedia Appendix 1 Search strategy and databases.

Multimedia Appendix 2 Extraction instrument.

## Abbreviations

ACB: African, Caribbean, and Black
ACB-NEWG: African, Caribbean, and Black National Expert Working Group
EGAP: Engagement, Governance, Access, and Protection
FNIGC: First Nations Information Governance Centre
HIC: high-income country
JBI: Joanna Briggs Institute
OCAP: Ownership, Control, Access, and Possession
PRISMA-P: Preferred Reporting Items for Systematic Reviews and Meta-Analysis Protocols
TM: thematic mapping
