# Supplementary material for: Determinants of Equitable Data Governance for African, Caribbean, and Black Communities in Health Research in High-Income Countries: Protocol for a Scoping Review
Source: JMIR Res Protoc. 2026 Feb 13;15:e82403. doi: 10.2196/82403 (PMC12949400; doi:10.2196/82403)
Supplement: Multimedia Appendix 1 [file resprot_v15i1e82403_app1.pdf]

Supplemental File 2: Search Strategy

**Database**	**Platform**	**Update**	**Date searched**
**MEDLINE**	Ovid	Ovid MEDLINE(R) ALL <1946 to December 16, 2024>	2024-12-17
**Embase**	Ovid	Embase Classic+Embase <1947 to 2024 December 16>	2024-12-17
**CINAHL**	EBSCO	1981 to present	2024-12-17
**APA PsycInfo**	Ovid	APA PsycInfo <1806 to December 2024 Week 2>	2024-12-17
**Scopus**	Scopus		2024-12-17

Medline (Ovid)

1 Data Management/ or Information Management/ or Health Information Management/

2 (data adj3 manag*).tw,kf.

3 information management.tw,kf.

4 or/1-3 [data management generally]

5 (data adj3 (govern or governing or governance)).tw,kf.

6 (data adj3 sovereign*).tw,kf.

7 (data adj3 steward*).tw,kf.

8 (data adj3 owns).tw,kf.

9 (data adj3 ownership).tw,kf.

10 (data adj3 custodian).tw,kf.

11 (data adj3 possess*).tw,kf.

12 ((FAIR adj3 (principle? or data)) or "findable accessible interoperable reusable").tw,kf.

13 (CARE principles adj10 data).tw. or CARE principles.kf.

14 ((Collective benefit* or "Authority to control") adj3 data).tw,kf.

15 OCAP.tw,kf.

16 (EGAP adj5 framework).tw,kf.

17 or/5-16 [governance principles]

18 information dissemination/

19	(data adj shar*).tw,kf.
20	(dashboard? or dash board?).tw,kf.
21	(Data adj2 harmon*).tw,kf.
22	((public or data) adj (deposit? or repositor*)).tw,kf.
23	or/18-22 [sharing]
24	(data adj3 priva*).tw,kf.
25	(data adj3 confidential*).tw,kf.
26	(data adj2 protect*).tw,kf.
27	(data adj2 security).tw,kf.
28	(data adj (store? or storage)).tw,kf.
29	(data adj (anonymi* or deidentif* or de identif*)).tw,kf.
30	or/24-29 [storage]
31	(data adj standard*).tw,kf.
32	(data adj (policy or policies)).tw,kf.
33	(data adj framework*).tw,kf.
34	or/31-33 [legal policy]
35	(data adj2 (equit* or inequit*)).tw,kf.
36	(data adj5 ethic*).tw,kf.
37	(data adj3 privacy).tw,kf.
38	(data adj3 responsib*).tw,kf.
39	data transparency.tw,kf.
40	Data accessibility.tw,kf.
41	or/35-40 [ethical aspects]
42	4 or 17 or 23 or 30 or 34 or 41 [all data management]
43	"Black or African American"/
44	Black People/
45	racial groups/
46	black?.ti,ab,kf.

47 afro*.ti,ab,kf.

48 (African adj (immigrant* or migrant* or ancestr*)).ti,ab,kf.

49 ((Angolan* or Beninois or Botswanan* or Burkinabe or Burkinese or Burundian* or Verdean* or Cameroonian* or "Central African Republican*" or Chadian* or Congolese or Ivorian* or Djiboutian* or Guinean* or Eritrea* or Eswatinian* or Ethiopian* or Gabonese or Gambian* or Ghanaian* or Kenyan* or Lesothoan* or Liberian* or Malagasy or Malawian* or Malian* or Mozambican* or Namibian* or Nigerien* or Nigerian* or Rwandan* or Senegalese or "Sierra Leonean*" or Somalian* or Sudanese or Tanzanian* or Togolese or Ugandan* or Zambian* or Zimbabwean*) adj3 (America or Andorra or Antigua or Aruba or Australia or Austria or Bahamas or Barbados or Barbuda or Belgium or Bermuda or Britain or Brunei or Bulgaria or Canada or Cayman Islands or Chile or Croatia or Curacao or Cyprus or Czechia or Denmark or Estonia or Finland or France or Germany or Gibraltar or Greenland or Greece or Guyana or Hong Kong or Hungary or Iceland or Ireland or Israel or Italy or Japan or Korea or Kuwait or Latvia or Liechtenstein or Lithuania or Luxembourg or Macao or Malta or Monaco or Nauru or Netherlands or New Zealand or Norway or Oman or Palau or Panama or Poland or Portugal or Romania or Russia or Saint Kitts or Nevis or San Marino or Scotland or Seychelles or Singapore or Slovakia or Slovenia or Spain or Sweden or Switzerland or Taiwan or "Trinidad and Tobago" or "Turks and Caicos" or United Arab Emirates or United Kingdom or Uruguay or Wales)).ti,ab,kf.

50 ((African* or Angolan* or Beninois or Botswanan* or Burkinabe or Burkinese or Burundian* or Verdean* or Cameroonian* or "Central African Republican*" or Chadian* or Congolese or Ivorian* or Djiboutian* or Guinean* or Eritrea* or Eswatinian* or Ethiopian* or Gabonese or Gambian* or Ghanaian* or Kenyan* or Lesothoan* or Liberian* or Malagasy or Malawian* or Malian* or Mozambican* or Namibian* or Nigerien* or Nigerian* or Rwandan* or Senegalese or "Sierra Leonean*" or Somalian* or Sudanese or Tanzanian* or Togolese or Ugandan* or Zambian* or Zimbabwean*) adj (American? or Caribbean? or Andorrans or Antiguan? or Aruban? or Australian? or Austrian? or Bahamian? or Barbadian? or Barbudan? or Belgian? or Bermudan? or Brit? or British or Bruneian? or Bulgarian? or Canadian? or Caymanian? or Chilean? or Croatian? or Curacaon? or Cypreans or Czechian? or Dane? or Dutch or Emiratis or Estonian? or Finns or French or German? or Gibraltarian? or Greek? or Greenlander? or Guyanese or Hongkonger* or "Hong Kongese" or Hungarian? or Icelander? or Irish or Israeli? or Italian? or Japanese? or Kittitians or Korean? or Kuwaiti? or Latvian? or Liechtensteiner? or Lithuanian? or Luxembourger? or Macanese or Maltese or Monegasque or Nauruan? or Nevisian or New Zealander? or Norwegian? or Omanis or Palaun? or Panamanian? or Pole? or Portugese or Romanian? or Russian? or San Marinarian? or Scots or Seychellois or Singaporian? or Slovak? or Slovenia? or Spaniard? or Swede? or Swiss or Taiwanese or Tobagonians or Trinidadian or "Turks and Caicos Islanders" or Uruguayan? or Welsh)).ti,ab,kf.

51 (African? adj3 (America or Andorra or Antigua or Aruba or Australia or Austria or Bahamas or Barbados or Barbuda or Belgium or Bermuda or Britain or Brunei or Bulgaria or Canada or Cayman Islands or Chile or Croatia or Curacao or Cyprus or Czechia or Denmark or Estonia or Finland or France or Germany or Gibraltar or Greenland or Greece or Guyana or

Hong Kong or Hungary or Iceland or Ireland or Israel or Italy or Japan or Korea or Kuwait or Latvia or Liechtenstein or Lithuania or Luxembourg or Macao or Malta or Monaco or Nauru or Netherlands or New Zealand or Norway or Oman or Palau or Panama or Poland or Portugal or Romania or Russia or Saint Kitts or Nevis or San Marino or Scotland or Seychelles or Singapore or Slovakia or Slovenia or Spain or Sweden or Switzerland or Taiwan or "Trinidad and Tobago" or "Turks and Caicos" or United Arab Emirates or United Kingdom or Uruguay or Wales)).ti,ab,kf.

52 (African Nova Scotian? or Gullah).ti,ab,kf.

53 "ethnic and racial minorities"/

54 (minorit* adj4 (group* or communit*)).ti,ab,kf.

55 ((rac* or ethnic*) adj4 (communit* or group* or minorit* or discriminat*)).ti,ab,kf.

56 exp racism/

57 racis*.ti,ab,kf.

58 or/43-57 [all ACB in high income]

59 42 and 58

60 ("38567458" or "37775604" or "38087341" or "36527096" or "33937178" or "34536537" or "12297682").ui.

61 59 and 60

Embase (Ovid)

1 (data adj3 manag*).tw,kf.

2 information management.tw,kf.

3 or/1-2

4 (data adj3 (govern or governing or governance)).tw,kf.

5 (data adj3 sovereign*).tw,kf.

6 (data adj3 steward*).tw,kf.

7 (data adj3 owns).tw,kf.

8 (data adj3 ownership).tw,kf.

9 (data adj3 custodian).tw,kf.

10 (data adj3 possess*).tw,kf.

11 ((FAIR adj3 (principle? or data)) or "findable accessible interoperable reusable").tw,kf.

12 (CARE principles adj10 data).tw. or CARE principles.kf.

13 ((Collective benefit* or "Authority to control") adj3 data).tw,kf.

14 OCAP.tw,kf.

15 (EGAP adj5 framework).tw,kf.

16 or/4-15

17 information dissemination/

18 (data adj shar*).tw,kf.

19 (dashboard? or dash board?).tw,kf.

20 (Data adj2 harmon*).tw,kf.

21 ((public or data) adj (deposit? or repositor*)).tw,kf.

22 or/17-21

23 exp data protection/ or information security/ or information storage/

24 (data adj3 priva*).tw,kf.

25 (data adj3 confidential*).tw,kf.

26 (data adj2 protect*).tw,kf.

27 (data adj2 security).tw,kf.

28 (data adj (store? or storage)).tw,kf.

29 (data adj (anonymi* or deidentif* or de identif*)).tw,kf.

30 or/23-29

31 (data adj standard*).tw,kf.

32 (data adj (policy or policies)).tw,kf.

33 (data adj framework*).tw,kf.

34 or/31-33

35 (data adj2 (equit* or inequit*)).tw,kf.

36 (data adj5 ethic*).tw,kf.

37 (data adj3 responsib*).tw,kf.

38 data transparency.tw,kf.

39 Data accessibility.tw,kf.

40 or/35-39

41 or/3,16,22,30,34,40

42 exp Black person/

43 ancestry group/

44 black?.ti,ab,kf.

45 afro*.ti,ab,kf.

46 (African adj (immigrant* or migrant* or ancestr*)).ti,ab,kf.

47 ((Angolan* or Beninois or Botswanan* or Burkinabe or Burkinese or Burundian* or Verdean* or Cameroonian* or "Central African Republican*" or Chadian* or Congolese or Ivorian* or Djiboutian* or Guinean* or Eritrea* or Eswatinian* or Ethiopian* or Gabonese or Gambian* or Ghanaian* or Kenyan* or Lesothoan* or Liberian* or Malagasy or Malawian* or Malian* or Mozambican* or Namibian* or Nigerien* or Nigerian* or Rwandan* or Senegalese or "Sierra Leonean*" or Somalian* or Sudanese or Tanzanian* or Togolese or Ugandan* or Zambian* or Zimbabwean*) adj3 (America or Andorra or Antigua or Aruba or Australia or Austria or Bahamas or Barbados or Barbuda or Belgium or Bermuda or Britain or Brunei or Bulgaria or Canada or Cayman Islands or Chile or Croatia or Curacao or Cyprus or Czechia or Denmark or Estonia or Finland or France or Germany or Gibraltar or Greenland or Greece or Guyana or Hong Kong or Hungary or Iceland or Ireland or Israel or Italy or Japan or Korea or Kuwait or Latvia or Liechtenstein or Lithuania or Luxembourg or Macao or Malta or Monaco or Nauru or Netherlands or New Zealand or Norway or Oman or Palau or Panama or Poland or Portugal or Romania or Russia or Saint Kitts or Nevis or San Marino or Scotland or Seychelles or Singapore or Slovakia or Slovenia or Spain or Sweden or Switzerland or Taiwan or "Trinidad and Tobago" or "Turks and Caicos" or United Arab Emirates or United Kingdom or Uruguay or Wales)).ti,ab,kf.

48 ((African* or Angolan* or Beninois or Botswanan* or Burkinabe or Burkinese or Burundian* or Verdean* or Cameroonian* or "Central African Republican*" or Chadian* or Congolese or Ivorian* or Djiboutian* or Guinean* or Eritrea* or Eswatinian* or Ethiopian* or Gabonese or Gambian* or Ghanaian* or Kenyan* or Lesothoan* or Liberian* or Malagasy or Malawian* or Malian* or Mozambican* or Namibian* or Nigerien* or Nigerian* or Rwandan* or Senegalese or "Sierra Leonean*" or Somalian* or Sudanese or Tanzanian* or Togolese or Ugandan* or Zambian* or Zimbabwean*) adj (American? or Caribbean? or Andorrans or Antiguan? or Aruban? or Australian? or Austrian? or Bahamian? or Barbadian? or Barbudan? or Belgian? or Bermudan? or Brit? or British or Bruneian? or Bulgarian? or Canadian? or Caymanian? or Chilean? or Croatian? or Curacaon? or Cypreans or Czechian? or Dane? or Dutch or Emiratis or Estonian? or Finns or French or German? or Gibraltarian? or Greek? or Greenlander? or Guyanese or Hongkonger* or "Hong Kongese" or Hungarian? or Icelander? or Irish or Israeli? or Italian? or Japanese? or Kittitians or Korean? or Kuwaiti? or Latvian? or Liechtensteiner? or Lithuanian? or Luxembourger? or Macanese or Maltese or Monegasque or Nauruan? or Nevisian or New Zealander? or Norwegian? or Omanis or Palaun? or Panamanian?

or Pole? or Portugese or Romanian? or Russian? or San Marinarian? or Scots or Seychellois or Singaporian? or Slovak? or Slovenia? or Spaniard? or Swede? or Swiss or Taiwanese or Tobagonians or Trinidadian or "Turks and Caicos Islanders" or Uruguayan? or Welsh)).ti,ab,kf.

49 (African? adj3 (America or Andorra or Antigua or Aruba or Australia or Austria or Bahamas or Barbados or Barbuda or Belgium or Bermuda or Britain or Brunei or Bulgaria or Canada or Cayman Islands or Chile or Croatia or Curacao or Cyprus or Czechia or Denmark or Estonia or Finland or France or Germany or Gibraltar or Greenland or Greece or Guyana or Hong Kong or Hungary or Iceland or Ireland or Israel or Italy or Japan or Korea or Kuwait or Latvia or Liechtenstein or Lithuania or Luxembourg or Macao or Malta or Monaco or Nauru or Netherlands or New Zealand or Norway or Oman or Palau or Panama or Poland or Portugal or Romania or Russia or Saint Kitts or Nevis or San Marino or Scotland or Seychelles or Singapore or Slovakia or Slovenia or Spain or Sweden or Switzerland or Taiwan or "Trinidad and Tobago" or "Turks and Caicos" or United Arab Emirates or United Kingdom or Uruguay or Wales)).ti,ab,kf.

50 (African Nova Scotian? or Gullah).ti,ab,kf.

51 "ethnic or racial aspects"/

52 (minorit* adj4 (group* or communit*)).ti,ab,kf.

53 ((rac* or ethnic*) adj4 (communit* or group* or minorit* or discriminat*)).ti,ab,kf.

54 exp racism/

55 racis*.ti,ab,kf.

56 or/42-55

57 41 and 56

CINAHL (EBSCO)

S8 (S1 OR S2 OR S3 OR S4 OR S5) AND (S6 AND S7)

S7 S1 OR S2 OR S3 OR S4 OR S5

S6 (MH "Black Persons+") OR (MH "People of Color+") OR TI(black#) OR AB(black#) OR TI(afro*) OR (AB(afro*) OR TI((African N0 (immigrant* or migrant* or ancestr*))) OR AB((African N0 (immigrant* or migrant* or ancestr*))) OR TI((Angolan* or Beninois or Botswanan* or Burkinabe or Burkinese or Burundian* or Verdean* or Cameroonian* or "Central African Republican*" or Chadian* or Congolese or Ivorian* or Djiboutian* or Guinean* or Eritrea* or Eswatinian* or Ethiopian* or Gabonese or Gambian* or Ghanaian* or Kenyan* or Lesothoan* or Liberian* or Malagasy or Malawian* or Malian* or Mozambican* or Namibian* or Nigerien* or Nigerian* or Rwandan* or Senegalese or "Sierra Leonean*" or Somalian* or Sudanese or Tanzanian* or Togolese or Ugandan* or Zambian* or Zimbabwean*) N2 (America or Andorra or Antigua or Aruba or Australia or Austria or Bahamas or Barbados or Barbuda or

Belgium or Bermuda or Britain or Brunei or Bulgaria or Canada or "Cayman Islands" or Chile or Croatia or Curacao or Cyprus or Czechia or Denmark or Estonia or Finland or France or Germany or Gibraltar or Greenland or Greece or Guyana or "Hong Kong" or Hungary or Iceland or Ireland or Israel or Italy or Japan or Korea or Kuwait or Latvia or Liechtenstein or Lithuania or Luxembourg or Macao or Malta or Monaco or Nauru or Netherlands or "New Zealand" or Norway or Oman or Palau or Panama or Poland or Portugal or Romania or Russia or "Saint Kitts" or Nevis or "San Marino" or Scotland or Seychelles or Singapore or Slovakia or Slovenia or Spain or Sweden or Switzerland or Taiwan or "Trinidad and Tobago" or "Turks and Caicos" or "United Arab Emirates" or "United Kingdom" or Uruguay or Wales)) OR AB((Angolan* or Beninois or Botswanan* or Burkinabe or Burkinese or Burundian* or Verdean* or Cameroonian* or "Central African Republican*" or Chadian* or Congolese or Ivorian* or Djiboutian* or Guinean* or Eritrea* or Eswatinian* or Ethiopian* or Gabonese or Gambian* or Ghanaian* or Kenyan* or Lesothoan* or Liberian* or Malagasy or Malawian* or Malian* or Mozambican* or Namibian* or Nigerien* or Nigerian* or Rwandan* or Senegalese or "Sierra Leonean*" or Somalian* or Sudanese or Tanzanian* or Togolese or Ugandan* or Zambian* or Zimbabwean*) N2 (America or Andorra or Antigua or Aruba or Australia or Austria or Bahamas or Barbados or Barbuda or Belgium or Bermuda or Britain or Brunei or Bulgaria or Canada or "Cayman Islands" or Chile or Croatia or Curacao or Cyprus or Czechia or Denmark or Estonia or Finland or France or Germany or Gibraltar or Greenland or Greece or Guyana or "Hong Kong" or Hungary or Iceland or Ireland or Israel or Italy or Japan or Korea or Kuwait or Latvia or Liechtenstein or Lithuania or Luxembourg or Macao or Malta or Monaco or Nauru or Netherlands or "New Zealand" or Norway or Oman or Palau or Panama or Poland or Portugal or Romania or Russia or "Saint Kitts" or Nevis or "San Marino" or Scotland or Seychelles or Singapore or Slovakia or Slovenia or Spain or Sweden or Switzerland or Taiwan or "Trinidad and Tobago" or "Turks and Caicos" or "United Arab Emirates" or "United Kingdom" or Uruguay or Wales)) OR TI((African* or Angolan* or Beninois or Botswanan* or Burkinabe or Burkinese or Burundian* or Verdean* or Cameroonian* or "Central African Republican*" or Chadian* or Congolese or Ivorian* or Djiboutian* or Guinean* or Eritrea* or Eswatinian* or Ethiopian* or Gabonese or Gambian* or Ghanaian* or Kenyan* or Lesothoan* or Liberian* or Malagasy or Malawian* or Malian* or Mozambican* or Namibian* or Nigerien* or Nigerian* or Rwandan* or Senegalese or "Sierra Leonean*" or Somalian* or Sudanese or Tanzanian* or Togolese or Ugandan* or Zambian* or Zimbabwean*) N0 (American? or Caribbean? or Andorrans or Antiguan? or Aruban? or Australian? or Austrian? or Bahamian? or Barbadian? or Barbudan? or Belgian? or Bermudan? or Brit? or British or Bruneian? or Bulgarian? or Canadian? or Caymanian? or Chilean? or Croatian? or Curacaon? or Cypreans or Czechian? or Dane? or Dutch or Emiratis or Estonian? or Finns or French or German? or Gibraltarian? or Greek? or Greenlander? or Guyanese or Hongkonger* or "Hong Kongese" or Hungarian? or Icelander? or Irish or Israeli? or Italian? or Japanese? or Kittitians or Korean? or Kuwaiti? or Latvian? or Liechtensteiner? or Lithuanian? or Luxembourger? or Macanese or Maltese or Monegasque or Nauruan? or Nevisian or "New Zealander?" or Norwegian? or Omanis or Palaun? or Panamanian? or Pole? or Portugese or Romanian? or Russian? or "San Marinarian?" or Scots or Seychellois or Singaporian? or Slovak? or Slovenia? or Spaniard? or Swede? or Swiss or Taiwanese or Tobagonians or Trinidadian or "Turks and Caicos Islanders" or Uruguayan? or

Welsh)) OR AB((African* or Angolan* or Beninois or Botswanan* or Burkinabe or Burkinese or Burundian* or Verdean* or Cameroonian* or "Central African Republican*" or Chadian* or Congolese or Ivorian* or Djiboutian* or Guinean* or Eritrea* or Eswatinian* or Ethiopian* or Gabonese or Gambian* or Ghanaian* or Kenyan* or Lesothoan* or Liberian* or Malagasy or Malawian* or Malian* or Mozambican* or Namibian* or Nigerien* or Nigerian* or Rwandan* or Senegalese or "Sierra Leonean*" or Somalian* or Sudanese or Tanzanian* or Togolese or Ugandan* or Zambian* or Zimbabwean*) N0 (American? or Caribbean? or Andorrans or Antiguan? or Aruban? or Australian? or Austrian? or Bahamian? or Barbadian? or Barbudan? or Belgian? or Bermudan? or Brit? or British or Bruneian? or Bulgarian? or Canadian? or Caymanian? or Chilean? or Croatian? or Curacaon? or Cypreans or Czechian? or Dane? or Dutch or Emiratis or Estonian? or Finns or French or German? or Gibraltarian? or Greek? or Greenlander? or Guyanese or Hongkonger* or "Hong Kongese" or Hungarian? or Icelander? or Irish or Israeli? or Italian? or Japanese? or Kittitians or Korean? or Kuwaiti? or Latvian? or Liechtensteiner? or Lithuanian? or Luxembourger? or Macanese or Maltese or Monegasque or Nauruan? or Nevisian or "New Zealander?" or Norwegian? or Omanis or Palaun? or Panamanian? or Pole? or Portugese or Romanian? or Russian? or "San Marinarian?" or Scots or Seychellois or Singaporian? or Slovak? or Slovenia? or Spaniard? or Swede? or Swiss or Taiwanese or Tobagonians or Trinidadian or "Turks and Caicos Islanders" or Uruguayan? or Welsh)) OR TI(African# N2 (America or Andorra or Antigua or Aruba or Australia or Austria or Bahamas or Barbados or Barbuda or Belgium or Bermuda or Britain or Brunei or Bulgaria or Canada or "Cayman Islands" or Chile or Croatia or Curacao or Cyprus or Czechia or Denmark or Estonia or Finland or France or Germany or Gibraltar or Greenland or Greece or Guyana or "Hong Kong" or Hungary or Iceland or Ireland or Israel or Italy or Japan or Korea or Kuwait or Latvia or Liechtenstein or Lithuania or Luxembourg or Macao or Malta or Monaco or Nauru or Netherlands or "New Zealand" or Norway or Oman or Palau or Panama or Poland or Portugal or Romania or Russia or "Saint Kitts" or Nevis or "San Marino" or Scotland or Seychelles or Singapore or Slovakia or Slovenia or Spain or Sweden or Switzerland or Taiwan or "Trinidad and Tobago" or "Turks and Caicos" or "United Arab Emirates" or "United Kingdom" or Uruguay or Wales)) OR AB(African# N2 (America or Andorra or Antigua or Aruba or Australia or Austria or Bahamas or Barbados or Barbuda or Belgium or Bermuda or Britain or Brunei or Bulgaria or Canada or "Cayman Islands" or Chile or Croatia or Curacao or Cyprus or Czechia or Denmark or Estonia or Finland or France or Germany or Gibraltar or Greenland or Greece or Guyana or "Hong Kong" or Hungary or Iceland or Ireland or Israel or Italy or Japan or Korea or Kuwait or Latvia or Liechtenstein or Lithuania or Luxembourg or Macao or Malta or Monaco or Nauru or Netherlands or "New Zealand" or Norway or Oman or Palau or Panama or Poland or Portugal or Romania or Russia or "Saint Kitts" or Nevis or "San Marino" or Scotland or Seychelles or Singapore or Slovakia or Slovenia or Spain or Sweden or Switzerland or Taiwan or "Trinidad and Tobago" or "Turks and Caicos" or "United Arab Emirates" or "United Kingdom" or Uruguay or Wales)) OR TI(African Nova Scotian# or Gullah) OR AB(African Nova Scotian# or Gullah) OR TI(minorit* N3 (group* or communit*)) OR AB(minorit* N3 (group* or communit*)) OR TI((rac* or ethnic*) N3 (communit* or group* or minorit* or discriminat*)) OR AB((rac* or ethnic*) N3 (communit* or group* or minorit* or discriminat*)) OR (MH "Racism+") OR TI(racis*) OR AB(racis*)

S5 TI((data N1 (equit* or inequit*))) OR (AB((data N1 (equit* or inequit*))) OR TI((data N4 ethic*)) OR (AB((data N4 ethic*)) OR TI((data N2 responsib*)) OR (AB((data N2 responsib*)) OR TI("data transparency") OR (AB("data transparency") OR TI("Data accessibility") OR (AB("Data accessibility")

S4 (MH "Data Security") OR TI((data N2 priva*)) OR (AB((data N2 priva*)) OR TI((data N2 confidential*)) OR (AB((data N2 confidential*)) OR TI((data N1 protect*)) OR (AB((data N1 protect*)) OR TI((data N1 security)) OR (AB((data N1 security)) OR TI((data N0 (store# or storage))) OR (AB((data N0 (store# or storage))) OR TI((data N0 (anonymi* or deidentif* or "de identif*"))) OR (AB((data N0 (anonymi* or deidentif* or "de identif*"))) OR TI((data N0 standard*)) OR (AB((data N0 standard*)) OR TI((data N0 (policy or policies))) OR (AB((data N0 (policy or policies))) OR TI((data N0 framework*)) OR (AB((data N0 framework*))

S3 TI((data N0 shar*)) OR (AB((data N0 shar*)) OR TI((dashboard# or "dash board#")) OR (AB((dashboard# or "dash board"#)) OR TI((Data N1 harmon*)) OR (AB((Data N1 harmon*)) OR TI(((public or data) N0 (deposit# or repositor*))) OR (AB(((public or data) N0 (deposit# or repositor*)))

S2 TI((data N2 (govern or governing or governance))) OR (AB((data N2 (govern or governing or governance))) OR TI((data N2 sovereign*)) OR (AB((data N2 sovereign*)) OR TI((data N2 steward*)) OR (AB((data N2 steward*)) OR TI((data N2 owns)) OR (AB((data N2 owns)) OR TI((data N2 ownership)) OR (AB((data N2 ownership)) OR TI((data N2 custodian)) OR (AB((data N2 custodian)) OR TI((data N2 possess*)) OR (AB((data N2 possess*)) OR TI(((FAIR N2 (principle# or data)) or "findable accessible interoperable reusable")) OR (AB(((FAIR N2 (principle# or data)) or "findable accessible interoperable reusable")) OR TI("CARE principles" N9 data) OR (AB("CARE principles" N9 data) OR TI((("Collective benefit*" or "Authority to control") N2 data)) OR (AB((("Collective benefit*" or "Authority to control") N2 data)) OR TI(OCAP) OR (AB(OCAP) OR TI((EGAP N4 framework)) OR (AB((EGAP N4 framework))

S1 (MH "Data Management") OR (MH "Health Information Management") OR (MH "Information Management") OR TI((data N2 manag*)) OR (AB((data N2 manag*)) OR TI("information management") OR (AB("information management")

APA PsycInfo (Ovid)

1 (data adj3 manag*).tw.

2 information management.tw.

3 or/1-2 [data management generally]

4 (data adj3 (govern or governing or governance)).tw.

5 (data adj3 sovereign*).tw.

6 (data adj3 steward*).tw.

7	(data adj3 owns).tw.
8	(data adj3 ownership).tw.
9	(data adj3 custodian).tw.
10	(data adj3 possess*).tw.
11	((FAIR adj3 (principle? or data)) or "findable accessible interoperable reusable").tw.
12	(CARE principles adj10 data).tw.
13	((Collective benefit* or "Authority to control") adj3 data).tw.
14	OCAP.tw.
15	(EGAP adj5 framework).tw.
16	or/4-15 [governance principles]
17	information dissemination/ or data sharing/ or open data/
18	(data adj shar*).tw.
19	(dashboard? or dash board?).tw.
20	(Data adj2 harmon*).tw.
21	((public or data) adj (deposit? or repositor*)).tw.
22	or/17-21 [sharing]
23	Information Security/
24	(data adj3 priva*).tw.
25	(data adj3 confidential*).tw.
26	(data adj2 protect*).tw.
27	(data adj2 security).tw.
28	(data adj (store? or storage)).tw.
29	(data adj (anonymi* or deidentif* or de identif*)).tw.
30	or/23-29 [storage]
31	(data adj standard*).tw.
32	(data adj (policy or policies)).tw.
33	(data adj framework*).tw.
34	or/30-33 [legal policy]

35 (data adj2 (equit* or inequit*)).tw.

36 (data adj5 ethic*).tw.

37 (data adj3 responsib*).tw.

38 data transparency.tw.

39 Data accessibility.tw.

40 or/35-39 [ethical aspects]

41 3 or 16 or 22 or 30 or 34 or 40 [all data management]

42 Black People/ or "people of color"/

43 "Racial and Ethnic Groups"/

44 black?.ti,ab.

45 afro*.ti,ab.

46 (African adj (immigrant* or migrant* or ancestr*)).ti,ab.

47 ((Angolan* or Beninois or Botswanan* or Burkinabe or Burkinese or Burundian* or Verdean* or Cameroonian* or "Central African Republican*" or Chadian* or Congolese or Ivorian* or Djiboutian* or Guinean* or Eritrea* or Eswatinian* or Ethiopian* or Gabonese or Gambian* or Ghanaian* or Kenyan* or Lesothoan* or Liberian* or Malagasy or Malawian* or Malian* or Mozambican* or Namibian* or Nigerien* or Nigerian* or Rwandan* or Senegalese or "Sierra Leonean*" or Somalian* or Sudanese or Tanzanian* or Togolese or Ugandan* or Zambian* or Zimbabwean*) adj3 (America or Andorra or Antigua or Aruba or Australia or Austria or Bahamas or Barbados or Barbuda or Belgium or Bermuda or Britain or Brunei or Bulgaria or Canada or Cayman Islands or Chile or Croatia or Curacao or Cyprus or Czechia or Denmark or Estonia or Finland or France or Germany or Gibraltar or Greenland or Greece or Guyana or Hong Kong or Hungary or Iceland or Ireland or Israel or Italy or Japan or Korea or Kuwait or Latvia or Liechtenstein or Lithuania or Luxembourg or Macao or Malta or Monaco or Nauru or Netherlands or New Zealand or Norway or Oman or Palau or Panama or Poland or Portugal or Romania or Russia or Saint Kitts or Nevis or San Marino or Scotland or Seychelles or Singapore or Slovakia or Slovenia or Spain or Sweden or Switzerland or Taiwan or "Trinidad and Tobago" or "Turks and Caicos" or United Arab Emirates or United Kingdom or Uruguay or Wales)).ti,ab.

48 ((African* or Angolan* or Beninois or Botswanan* or Burkinabe or Burkinese or Burundian* or Verdean* or Cameroonian* or "Central African Republican*" or Chadian* or Congolese or Ivorian* or Djiboutian* or Guinean* or Eritrea* or Eswatinian* or Ethiopian* or Gabonese or Gambian* or Ghanaian* or Kenyan* or Lesothoan* or Liberian* or Malagasy or Malawian* or Malian* or Mozambican* or Namibian* or Nigerien* or Nigerian* or Rwandan* or Senegalese or "Sierra Leonean*" or Somalian* or Sudanese or Tanzanian* or Togolese or Ugandan* or Zambian* or Zimbabwean*) adj (American? or Caribbean? or Andorrans or

Antiguan? or Aruban? or Australian? or Austrian? or Bahamian? or Barbadian? or Barbudan? or Belgian? or Bermudan? or Brit? or British or Bruneian? or Bulgarian? or Canadian? or Caymanian? or Chilean? or Croatian? or Curacaon? or Cypreans or Czechian? or Dane? or Dutch or Emiratis or Estonian? or Finns or French or German? or Gibraltarian? or Greek? or Greenlander? or Guyanese or Hongkonger* or "Hong Kongese" or Hungarian? or Icelander? or Irish or Israeli? or Italian? or Japanese? or Kittitians or Korean? or Kuwaiti? or Latvian? or Liechtensteiner? or Lithuanian? or Luxembourger? or Macanese or Maltese or Monegasque or Nauruan? or Nevisian or New Zealander? or Norwegian? or Omanis or Palaun? or Panamanian? or Pole? or Portugese or Romanian? or Russian? or San Marinarian? or Scots or Seychellois or Singaporian? or Slovak? or Slovenia? or Spaniard? or Swede? or Swiss or Taiwanese or Tobagonians or Trinidadian or "Turks and Caicos Islanders" or Uruguayan? or Welsh)).ti,ab.

49 (African? adj3 (America or Andorra or Antigua or Aruba or Australia or Austria or Bahamas or Barbados or Barbuda or Belgium or Bermuda or Britain or Brunei or Bulgaria or Canada or Cayman Islands or Chile or Croatia or Curacao or Cyprus or Czechia or Denmark or Estonia or Finland or France or Germany or Gibraltar or Greenland or Greece or Guyana or Hong Kong or Hungary or Iceland or Ireland or Israel or Italy or Japan or Korea or Kuwait or Latvia or Liechtenstein or Lithuania or Luxembourg or Macao or Malta or Monaco or Nauru or Netherlands or New Zealand or Norway or Oman or Palau or Panama or Poland or Portugal or Romania or Russia or Saint Kitts or Nevis or San Marino or Scotland or Seychelles or Singapore or Slovakia or Slovenia or Spain or Sweden or Switzerland or Taiwan or "Trinidad and Tobago" or "Turks and Caicos" or United Arab Emirates or United Kingdom or Uruguay or Wales)).ti,ab.

50 (African Nova Scotian? or Gullah).ti,ab.

51 "Race (Anthropological)"/

52 (minorit* adj4 (group* or communit*)).ti,ab.

53 ((rac* or ethnic*) adj4 (communit* or group* or minorit* or discriminat*)).ti,ab.

54 "Race and Ethnic Discrimination"/

55 racis*.ti,ab.

56 or/42-55 [all ACB in high income]

57 41 and 56

Scopus

(TITLE-ABS-KEY ((data W/2 manag*) OR "information management") OR TITLE-ABS-KEY ((data W/2 (govern OR governing OR governance)) OR (data W/2 sovereign*) OR (data W/2 steward*) OR (data W/2 owns) OR (data W/2 ownership) OR (data W/2 custodian) OR (data W/2 possess*) OR (fair W/2 (principle? OR data)) OR "findable accessible interoperable reusable" OR (("Collective benefit*" OR "Authority to control") W/2 data) OR ocap OR (egap W/4 framework)) OR TITLE-ABS ("CARE principles" W/9 data) OR KEY (

"CARE principles") OR TITLE-ABS-KEY ((data W/0 shar*) OR (dashboard? OR "dash board?") OR (data W/1 harmon*) OR ((public OR data) W/0 (deposit? OR repositor*))) OR TITLE-ABS-KEY ((data W/2 priva*) OR (data W/2 confidential*) OR (data W/1 protect*) OR (data W/1 security) OR (data W/0 (store? OR storage)) OR (data W/0 (anonymi* OR deidentif* OR "de identif*"))) OR TITLE-ABS-KEY ((data W/0 standard*) OR (data W/0 (policy OR policies)) OR (data W/0 framework*)) OR TITLE-ABS-KEY ((data W/1 (equit* OR inequit*)) OR (data W/4 ethic*) OR (data W/2 privacy) OR (data W/2 responsib*) OR "data transparency" OR "Data accessibility")) AND (TITLE-ABS-KEY (black? OR afro* OR (african W/0 (immigrant* OR migrant* OR ancestr*))) OR TITLE-ABS-KEY ((angolan* OR beninois OR botswanan* OR burkinabe OR burkinese OR burundian* OR verdean* OR cameroonian* OR "Central African Republican*" OR chadian* OR congolese OR ivorian* OR djiboutian* OR guinean* OR eritrea* OR eswatinian* OR ethiopian* OR gabonese OR gambian* OR ghanaian* OR kenyan* OR lesothoan* OR liberian* OR malagasy OR malawian* OR malian* OR mozambican* OR namibian* OR nigerien* OR nigerian* OR rwandan* OR senegalese OR "Sierra Leonean*" OR somalian* OR sudanese OR tanzanian* OR togolese OR ugandan* OR zambian* OR zimbabwean*) W/2 (america OR andorra OR antigua OR aruba OR australia OR austria OR bahamas OR barbados OR barbuda OR belgium OR bermuda OR britain OR brunei OR bulgaria OR canada OR "Cayman Islands" OR chile OR croatia OR curacao OR cyprus OR czechia OR denmark OR estonia OR finland OR france OR germany OR gibraltar OR greenland OR greece OR guyana OR "Hong Kong" OR hungary OR iceland OR ireland OR israel OR italy OR japan OR korea OR kuwait OR latvia OR liechtenstein OR lithuania OR luxembourg OR macao OR malta OR monaco OR nauru OR netherlands OR "New Zealand" OR norway OR oman OR palau OR panama OR poland OR portugal OR romania OR russia OR "Saint Kitts" OR nevis OR "San Marino" OR scotland OR seychelles OR singapore OR slovakia OR slovenia OR spain OR sweden OR switzerland OR taiwan OR "Trinidad and Tobago" OR "Turks and Caicos" OR "United Arab Emirates" OR "United Kingdom" OR uruguay OR wales)) OR TITLE-ABS-KEY ((african* OR angolan* OR beninois OR botswanan* OR burkinabe OR burkinese OR burundian* OR verdean* OR cameroonian* OR "Central African Republican*" OR chadian* OR congolese OR ivorian* OR djiboutian* OR guinean* OR eritrea* OR eswatinian* OR ethiopian* OR gabonese OR gambian* OR ghanaian* OR kenyan* OR lesothoan* OR liberian* OR malagasy OR malawian* OR malian* OR mozambican* OR namibian* OR nigerien* OR nigerian* OR rwandan* OR senegalese OR "Sierra Leonean*" OR somalian* OR sudanese OR tanzanian* OR togolese OR ugandan* OR zambian* OR zimbabwean*) W/0 (american? OR caribbean? OR andorrans OR antiguan? OR aruban? OR australian? OR austrian? OR bahamian? OR barbadian? OR barbudan? OR belgian? OR bermudan? OR brit? OR british OR bruneian? OR bulgarian? OR canadian? OR caymanian? OR chilean? OR croatian? OR curacaon? OR cypreans OR czechian? OR dane? OR dutch OR emiratis OR estonian? OR finns OR french OR german? OR gibraltarian? OR greek? OR greenlander? OR guyanese OR hongkonger* OR "Hong Kongese" OR hungarian? OR icelander? OR irish OR israeli? OR italian? OR japanese? OR kittitians OR korean? OR kuwaiti? OR latvian? OR liechtensteiner? OR lithuanian? OR luxembourger? OR macanese OR maltese OR monegasque OR nauruan? OR nevisian OR "New Zealander?" OR norwegian? OR omanis OR palaun? OR panamanian? OR pole? OR portugese OR romanian?

OR russian? OR "San Marinarian?" OR scots OR seychellois OR singaporian? OR slovak? OR slovenia? OR spaniard? OR swede? OR swiss OR taiwanese OR tobagonians OR trinidadian OR "Turks and Caicos Islanders" OR uruguayan? OR welsh)) OR TITLE-ABS-KEY (african? W/2 (america OR andorra OR antigua OR aruba OR australia OR austria OR bahamas OR barbados OR barbuda OR belgium OR bermuda OR britain OR brunei OR bulgaria OR canada OR "Cayman Islands" OR chile OR croatia OR curacao OR cyprus OR czechia OR denmark OR estonia OR finland OR france OR germany OR gibraltar OR greenland OR greece OR guyana OR "Hong Kong" OR hungary OR iceland OR ireland OR israel OR italy OR japan OR korea OR kuwait OR latvia OR liechtenstein OR lithuania OR luxembourg OR macao OR malta OR monaco OR nauru OR netherlands OR "New Zealand" OR norway OR oman OR palau OR panama OR poland OR portugal OR romania OR russia OR "Saint Kitts" OR nevis OR "San Marino" OR scotland OR seychelles OR singapore OR slovakia OR slovenia OR spain OR sweden OR switzerland OR taiwan OR "Trinidad and Tobago" OR "Turks and Caicos" OR "United Arab Emirates" OR "United Kingdom" OR uruguay OR wales)) OR TITLE-ABS-KEY (("African Nova Scotian?" OR gullah) OR (minorit* W/3 (group* OR communit*)) OR ((rac* OR ethnic*) W/3 (communit* OR group* OR minorit* OR discriminat*)) OR racis*))
