# Supplementary material for: Determinants of Equitable Data Governance for African, Caribbean, and Black Communities in Health Research in High-Income Countries: Protocol for a Scoping Review
Source: JMIR Res Protoc. 2026 Feb 13;15:e82403. doi: 10.2196/82403 (PMC12949400; doi:10.2196/82403)
Supplement: Multimedia Appendix 2 [file resprot_v15i1e82403_app2.pdf]

Supplemental File 3: Data extraction instrument

Table 1. Extraction dimensions

Dimensions	Details	Additional explanation as applicable
General information/Source	Authors, year, country of study, language, funder	
Study characteristics	Aim of the study, study design, data source, methods, target population, ACB involvement, Aim of intervention, definition of concepts	
Study participants	Description of study population, characteristics of study participants, recruitment strategies, sampling and sample size, age	
Implementation strategies or intervention	Description of the intervention in relation to the health research partnerships, types of interventions	Organizational involvement (research partnerships, data management, knowledge mobilization in addressing ACB health needs).
Data analysis	Identify and code the activities or intervention strategies and categorise them according to the 5 key organizational capacity (resources, infrastructure, knowledge & skills, culture & climate, engagement & partnership) in addition to any other approach that does not fall within the categories.	
Outcome details	increased in community awareness on health research, lead research, genuine involvement, empowerment, data management, ownership, changes in	For the outcomes, data will be extracted for each outcome as applicable and

Dimensions	Details	Additional explanation as applicable
	societal structures, evidence-based policies and legislations	categorised using the five principles as described.
Equity dimensions	Dimensions using variables such as (place, religion, occupation, gender, race, education, social capital, and socio-economic status) as applicable	Any variables considered in the intervention population and/or groups—place (rural or urban setting), occupation, education, income status that addresses social determinants of health.
Context characteristics	ACB-led organizations, ACB communities, Community leaders	Description of ACB organizations

Table 2. Preliminary extraction sheet

ID	Author(s)/Year	Title	Objective of the study/Research question	Study Country of focus	Health Research Specificity	Study Population	Demographic characteristics of included study population (Foreign-born immigrant and Non migrant of their country of residence)	Key Study Design (Quan, Qual or Mixed)	Data analysis method: Qual (Thematic content..) Quan (Uni, Bi variate..)
